# Alpha-ketoglutarate suppresses the NF-κB-mediated inflammatory pathway and enhances the PXR-regulated detoxification pathway

**DOI:** 10.18632/oncotarget.16875

**Published:** 2017-04-06

**Authors:** Liuqin He, Huan Li, Niu Huang, Xihong Zhou, Junquan Tian, Tiejun Li, Jing Wu, Yanan Tian, Yulong Yin, Kang Yao

**Affiliations:** ^1^ Key Laboratory of Agro-Ecological Processes in Subtropical Region, Institute of Subtropical Agriculture, Chinese Academy of Sciences, Scientific Observing and Experimental Station of Animal Nutrition and Feed Science in South-Central, Ministry of Agriculture, Hunan Provincial Engineering Research Center for Healthy Livestock and Poultry Production, Changsha, Hunan 410125, China; ^2^ University of Chinese Academy of Sciences, Huairou, Beijing 10008, China; ^3^ College of Animal Science and Technology, Hunan Agricultural University, Hunan, Changsha 410128, China; ^4^ Hunan Co-Innovation Center of Animal Production Safety, Hunan, Changsha 410128, China; ^5^ Department of Veterinary Physiology and Pharmacology, Texas A&M University, College Station, TX 77843, USA

**Keywords:** AKG, pregnane X receptor, NF-κB, CYP450, intestinal inflammation

## Abstract

Alpha-ketoglutarate (AKG) is a critical nutritional factor in the maintenance of intestinal homeostasis. However, the relative mechanism of AKG has not been well understood. It was recently shown that the interaction between nuclear factor kappa B (NF-κB)-mediated inflammatory pathway and pregnane X receptor (PXR)-regulated detoxification pathway is a check and balance mechanism for keeping the homeostatic state of the intestine, preventing the onset of intestinal inflammation which may lead to cancer. In the current study we used lipopolysaccharide (LPS)-challenged piglet and intestinal porcine epithelial cells-J2 models to investigate the effects of dietary AKG supplementation on the intestinal immune system and PXR regulated target expression. We found that LPS induced significant activation of the NF-κB-mediated inflammatory pathway with concomitant impairment of intestinal nutrient absorption. AKG administration increased intracellular AKG and its metabolite concentrations and enhanced the mRNA expression of alpha-ketoglutarate dehydrogenase *in vivo* and *in vitro*. Thus dietary AKG supplementation reversed the adverse effects induced by LPS. We also found a strong inhibitory effects on the NF-κB-mediated inflammatory pathway, especially, in the AKG-treated intestinal tissues, LPS-induced NF-κB phosphorylation was inhibited and TNF-α was suppressed. Interestingly, AKG has potent effects in regulating the PXR and its downstream targets such as CYP3As and CYP2Bs *in vivo* and *in vitro*, although AKG is not a known PXR ligand. One potential mechanism for the up-regulation of the PXR pathway is through the down-regulation of NF-κB pathway which in turn de-represses the PXR-regulated target expression. Taken together, our results suggest that AKG improves intestinal immune system through modulating the interaction between PXR and NF-κB. Our findings have important implications for the prevention and treatment of intestinal inflammatory diseases in neonates.

## INTRODUCTION

The gut is a complex organ and its health is maintained through intricate interaction between nutrients, commensal microbiota and host intestinal epithelium [1]. As an organ that constantly comes into contact with the ingested nutrients together with the inevitable toxic substances, numerous commensal bacteria flora [2], the gut epithelium has various detoxification mechanisms involved in many nuclear receptors (NRs). NRs (*e.g.,* pregnane X receptor (PXR), constitutive androstane receptor (CAR), retinoic X receptor (RXR)) are a growing family of regulatory factors that exert gut homeostatic at the interface between nutrient metabolism and gut-associated immunity [3]. Recently, the xenobiotic receptors PXR has been reported to play an important role in the maintenance of gut homeostasis [4, 5]. Gu et al. found that the expression of nuclear factor kappa B (NF-κB) was increased in DSS-induced colitis of the PXR null mice [6]. Kusunoki et al. demonstrated that the p65 subunit of NF-κB also interacts with the PXR partner RXRα to regulate cytochrome P450 (CYP450) activity, and this interaction may account for the inhibition of drug metabolism observed in inflammatory states [7]. This suggest that the bidirectional negative crosstalk between PXR and NF-κB pathway is important for the health of gut epithelium and xenobiotic metabolism [8].

To date, numerous clinical studies have reported the importance of PXR as a drug target especially in the treatment of liver disease. However, whether nutritional intervention (*e.g.,* alpha-ketoglutarate, amino acids) could improve PXR-regulated detoxification pathway to alleviate intestinal inflammatory response remains largely unknown. Alpha-ketoglutarate (AKG) is of critical nutritional factor in the maintenance of gut homeostasis [9]. It served as a precursors of glutamine and glutamate in tissues and has shown clinical benefit that improve immunity in malnourished subjects or inflammatory diseases [9, 10]. However, the mechanistic role of AKG has not been well understood. It was recently shown that the interaction between NF-κB-mediated inflammatory pathway and PXR-regulated detoxification pathway is a check and balance mechanism for keeping the homeostatic state of the intestine, preventing the onset of intestinal inflammation which may lead to cancer [11]. In the intestinal mucosa, to determine the hypothesis that whether AKG could potentially modulate PXR pathway, we performed *in vitro* preliminary study of the effect of AKG metabolism on the PXR activation. The results showed that in the PXR overexpression enterocyte, the expression of alpha-ketoglutarate dehydrogenase (OGDH) involved in tricarboxylic acid (TCA) cycle was remarkably upregulated, suggesting that there was a strong correlation between the PXR activation and AKG metabolism. Therefore, the present study was conducted to further test the hypothesis that AKG improves intestinal immune system through suppressing the NF-κB-mediated inflammatory pathway and enhancing the PXR-regulated detoxification pathway in the piglet intestinal inflammatory model.

## RESULTS

### Serum biochemical parameters

**Figure 1:**
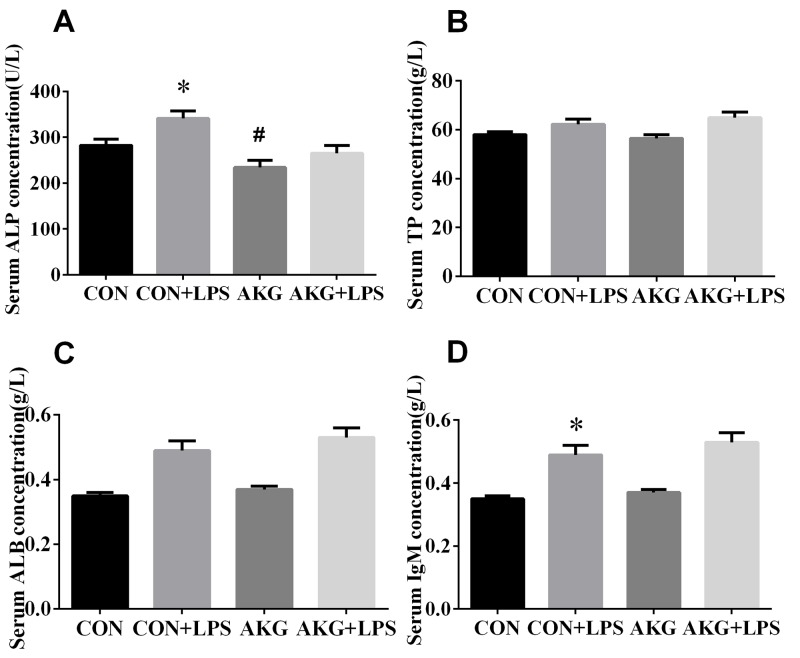
Effects of AKG supplementation on serum biochemical parameters of weaned piglets Values are LSmean plus pooled SEM, n=8. ^*^Indicates a statistically significant difference for challenge (saline or LPS) (P < 0.05). ^#^Indicates a statistically significant difference for dietary treatment (basal or AKG) (P < 0.05).

To explain the physiological effects of AKG on the weaned piglets, The serum levels of biochemical parameters were determined as a reflection of the metabolism and visceral organ status of pigs in response to *Escherichia coli* lipopolysaccharide (*E.coli* LPS) challenge (Figure 1). The results showed that neither diets nor LPS challenge affected (P > 0.05) the concentration of serum total protein (TP) (Figure 1B) and total albumin (ALB) (Figure 1C) by pigs. However, compared with pigs fed the basal diet, the concentration of alkaline phosphatase (ALP) (Figure 1A) in pigs fed the AKG diet decreased (P<0.05) by 16.8 %. Moreover, LPS challenge remarkably increased (P < 0.05) the concentrations of ALP and immunoglobulin M (IgM) (Figure 1D) in piglets both the basal diet and AKG diet. There is no LPS challenge × diet interaction effect (P < 0.05) in the concentrations of serum variables.

### Serum inflammatory cytokines

**Figure 2:**
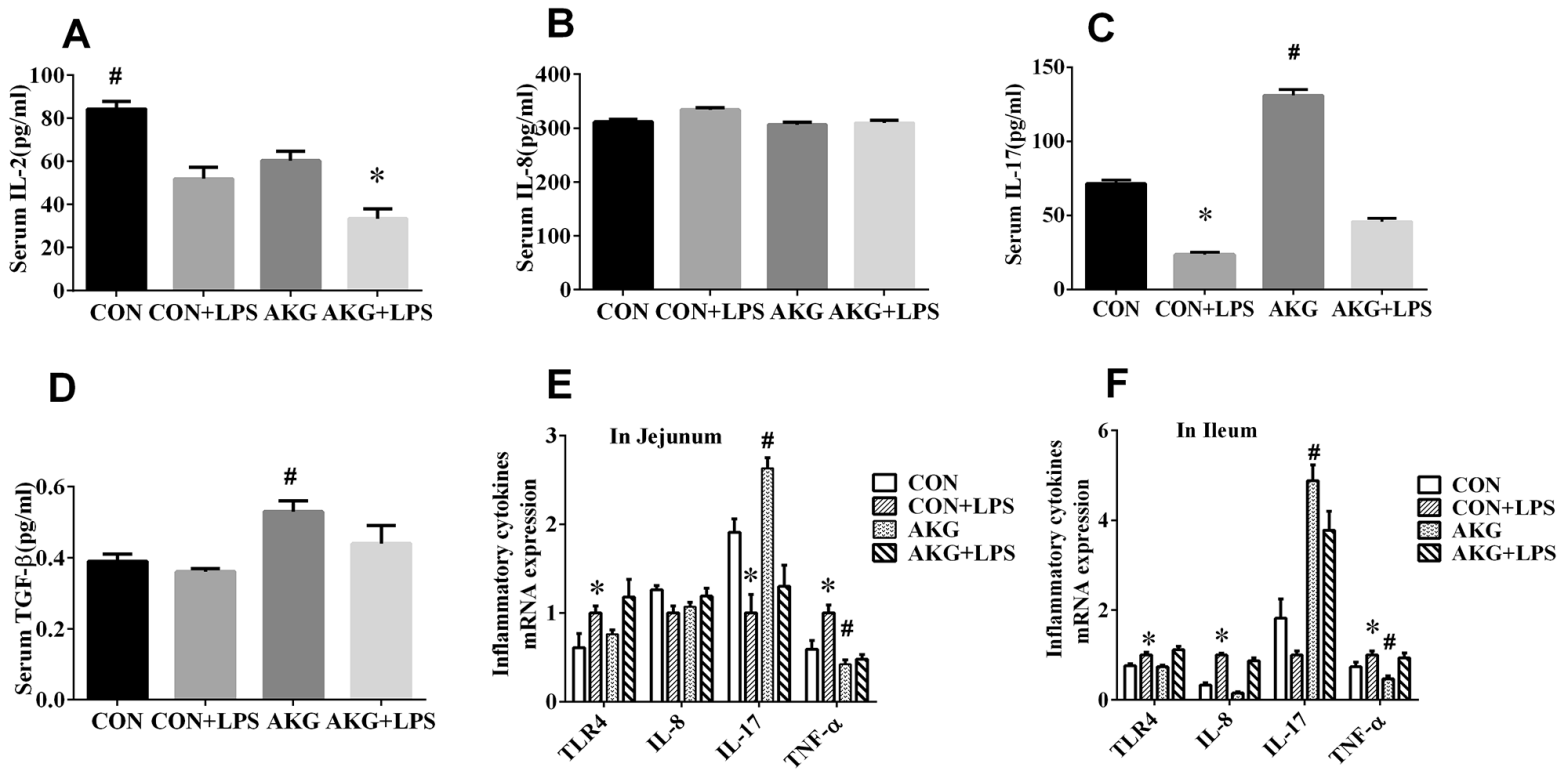
Effects of AKG supplementation on serum concentration and mRNA expression of inflammatory cytokines in weaned piglets

Inflammatory cytokines comprise a major immune defense system for preventing organ injury in response to inflammatory stimulus, such as interleukin 2(IL-2), interleukin 8(IL-8), interleukin 10(IL-10), interleukin 17(IL-17), and transforming growth factor–β (TGF-β). Neither diets nor LPS challenge affected serum IL-8 (Figure 2B) concentration AKG supplementation significantly reduced (P < 0.05) the concentration of IL-2(Figure 2A) by 18.3%, but remarkably enhanced (P < 0.05) the content of IL-17(Figure 2C) and TGF-β (Figure 2D) by 83.5% and 35.9%, respectively, when compared with the basal diet. Furthermore, serum concentration of IL-2 and IL-17 were greatly affected (P < 0.05) by LPS challenge. No LPS challenge × diet interaction was observed in the secretory of serum inflammatory cytokines. This results indicated that LPS stimulated the secretory of inflammatory cytokines in the weaned piglets, while the addition of AKG improves anti-inflammatory cytokines to defend the inflammation.

### Serum AKG and its metabolites

**Figure 3:**
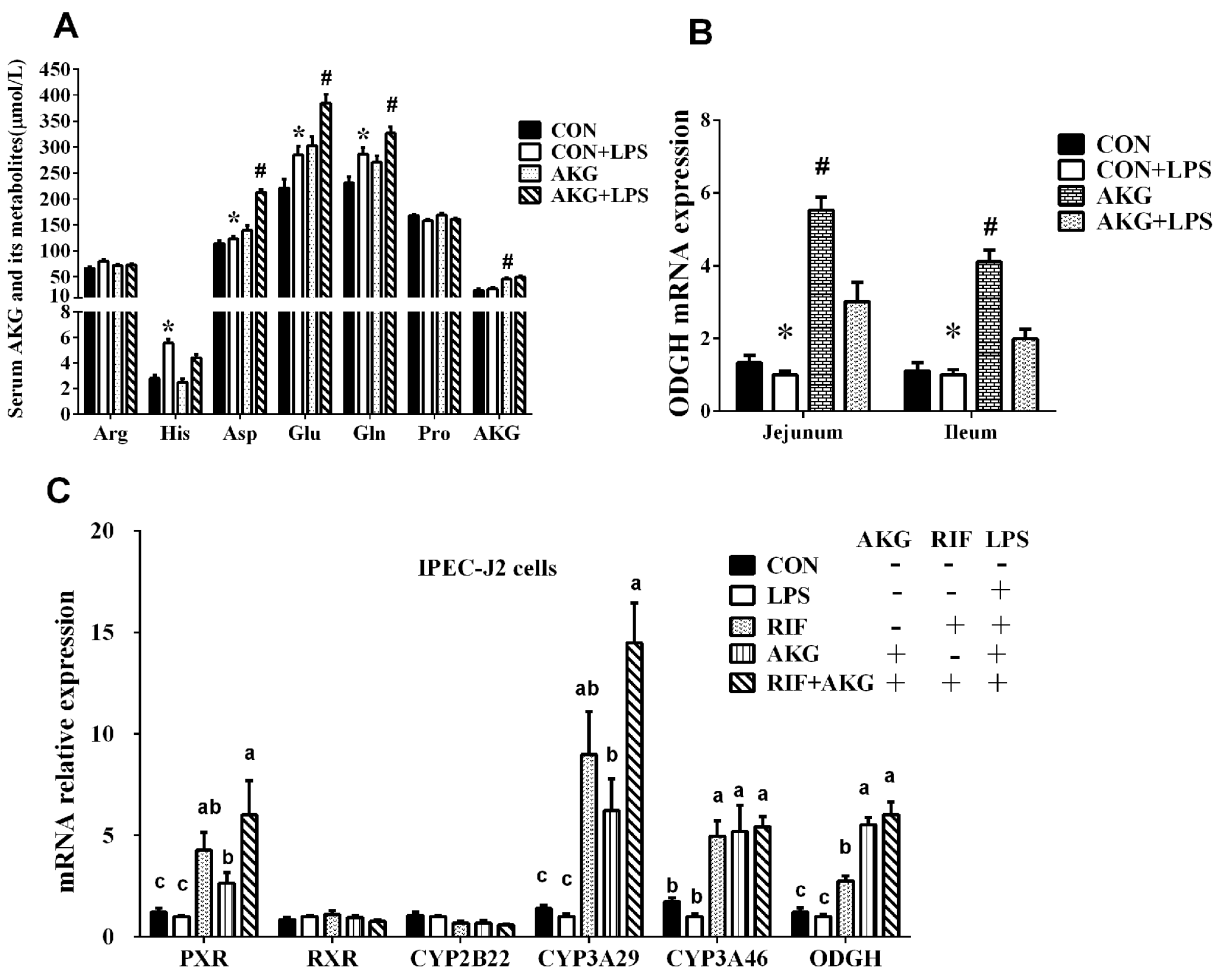
Effects of AKG supplementation on serum AKG and its metabolite concentrations, the mRNA expression of OGDH and PXR-regulated detoxification pathway **(A)** The concentrations of serum AKG and its metabolites. **(B)** The mRNA expression of OGDH in the jejunum and ileum of weaned piglets. **(C)** The mRNA expression of ODGH and PXR-regulated detoxification pathway in the IPEC-J2 cells. Different small letter superscripts represent significant difference (P < 0.05).

Amino acids play vital roles as metabolic intermediates in nutrition and immune response. To investigate whether impaired intestinal nutrient absorption contributes to inflammation in piglets, we examined some amino acids related to AKG metabolism in serum (Figure 3A). The data showed that the concentration of L-histidine (His), L-aspartate (Asp), L-glutamate (Glu), and L-glutamine (Gln) significantly increased (P <0.05) by LPS challenge. Especially, serum Asp, Glu, Gln, and proline (Pro) contents in pigs fed the AKG diet were higher (P <0.05) than pigs fed the basal diet. There is no LPS challenge × diet interaction effect (P < 0.05) in the concentrations of AKG and its metabolites. This suggests that AKG administration could increase the concentrations of serum AKG and its metabolites by desamidization and transamidation.

### Expression of NF-κB pathway

**Figure 4:**
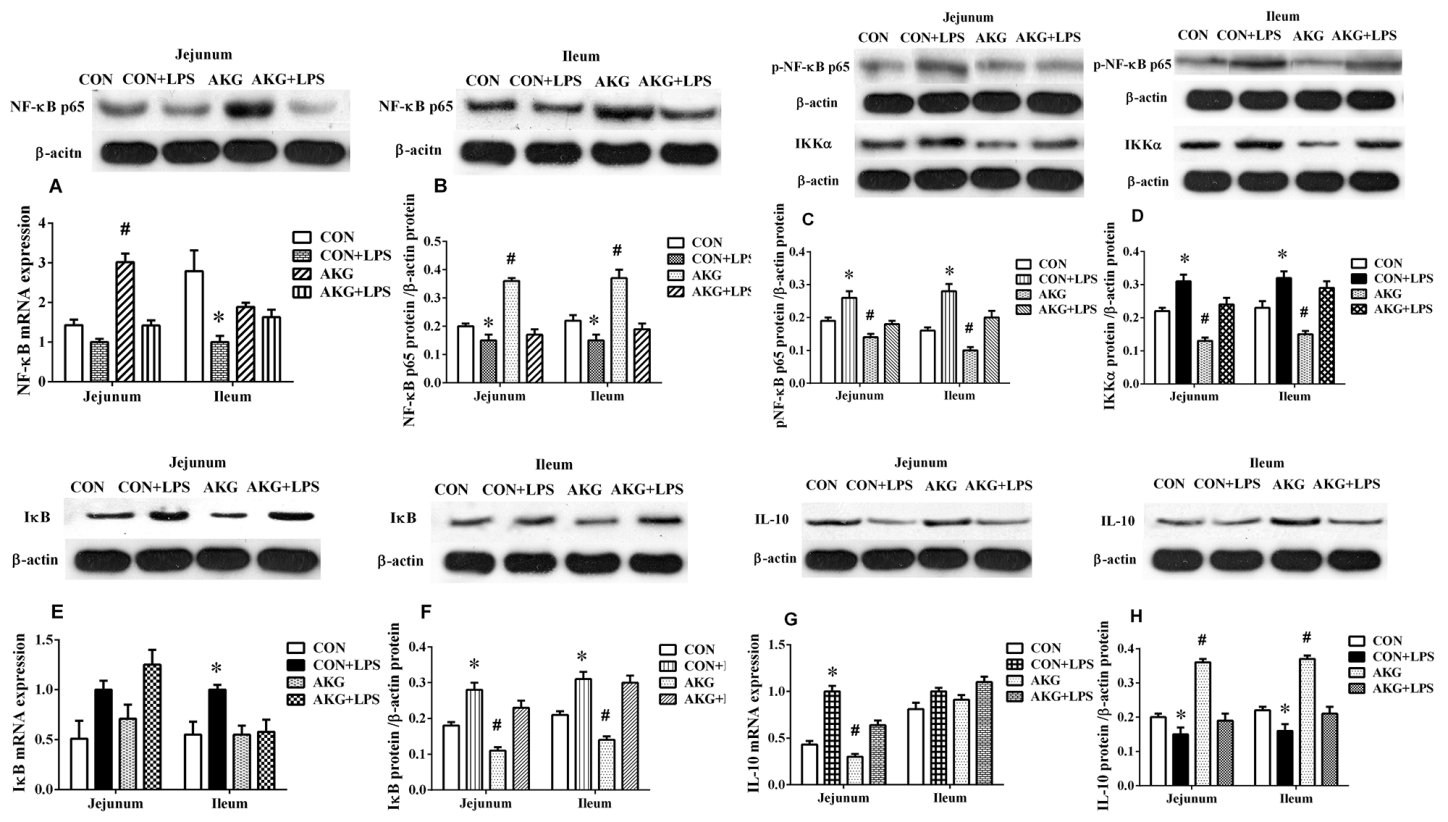
Effects of AKG supplementation on the expression of the NF-κB-mediated inflammatory pathway in the jejunum and ileum of weaned piglets

To validate that AKG was driving the anti-inflammatory response *in vivo*, we induced piglets intestinal inflammation using the intraperitoneal administration of *E.coli* LPS. The primary focus was to monitor the expression of NF-κB pathway using the real-time qPCR technique. In the jejunum, either diets or LPS challenge increased (P < 0.05) the mRNA expression of tumor necrosis factor-α (TNF-α) (Figure 2E) and IL-10 (Figure 4G) by piglets. However, AKG supplementation decreased the mRNA level of TNF-α as well as IL-10 in the jejunum. Moreover, LPS challenge remarkably increased the mRNA expression of Toll-like Receptor 4 (TLR4) (Figure 2E), while decreased IL-17 mRNA abundance (Figure 2E). In the ileum, compared with the saline-treated pigs, the mRNA levels of TLR4 (Figure 2F), IL-8(Figure 2F), and TNF-α (Figure 2F) were significantly increased (P<0.05)in the LPS-challenged piglets, while IL-17 mRNA abundance was significantly decreased. In addition, LPS challenge increased the mRNA expression of inhibitor of nuclear factor kappa-B (IκB) (Figure 4E) in the jejunum and ileum, while decreasing NF-κB mRNA level (Figure 4A). Compared with the pigs fed the basal diet, the mRNA levels of NF-κB (Figure 4A) in pigs fed the AKG diet were increased in the jejunum.

To further investigate whether AKG activation sensitizes piglets to the NF-κB-mediated inflammatory pathway, we determined the expression of the NF-κB pathway related key proteins using the Western blot technique. The results showed that LPS challenge increased (P < 0.05) the phosphorylation expression level of NF-κBp65 protein (Figure 4C), while decreased (P < 0.05) the expression of NF-κB p65protein (Figure 4B) in the jejunum and ileum of piglets fed the basal and AKG diets. Dietary supplementation with AKG increased (P < 0.05) the expression of NF-κBp65 and IL-10 (Figure 4H) proteins in the jejunum and ileum of both saline- and LPS-treated piglets but decreased the expression of phosphorylated NF-κBp65, inhibitor of nuclear factor kappa-B kinase α(IKKα) (Figure 4D) and IκB (Figure 4F) proteins. These results suggest that the LPS-challenged piglets are significantly susceptible to AKG mediated changes in NF-κB pathway, in which corroborate previous observations that AKG protects against LPS mediated NF-κB pathway.

### Expression of PXR pathway

**Figure 5:**
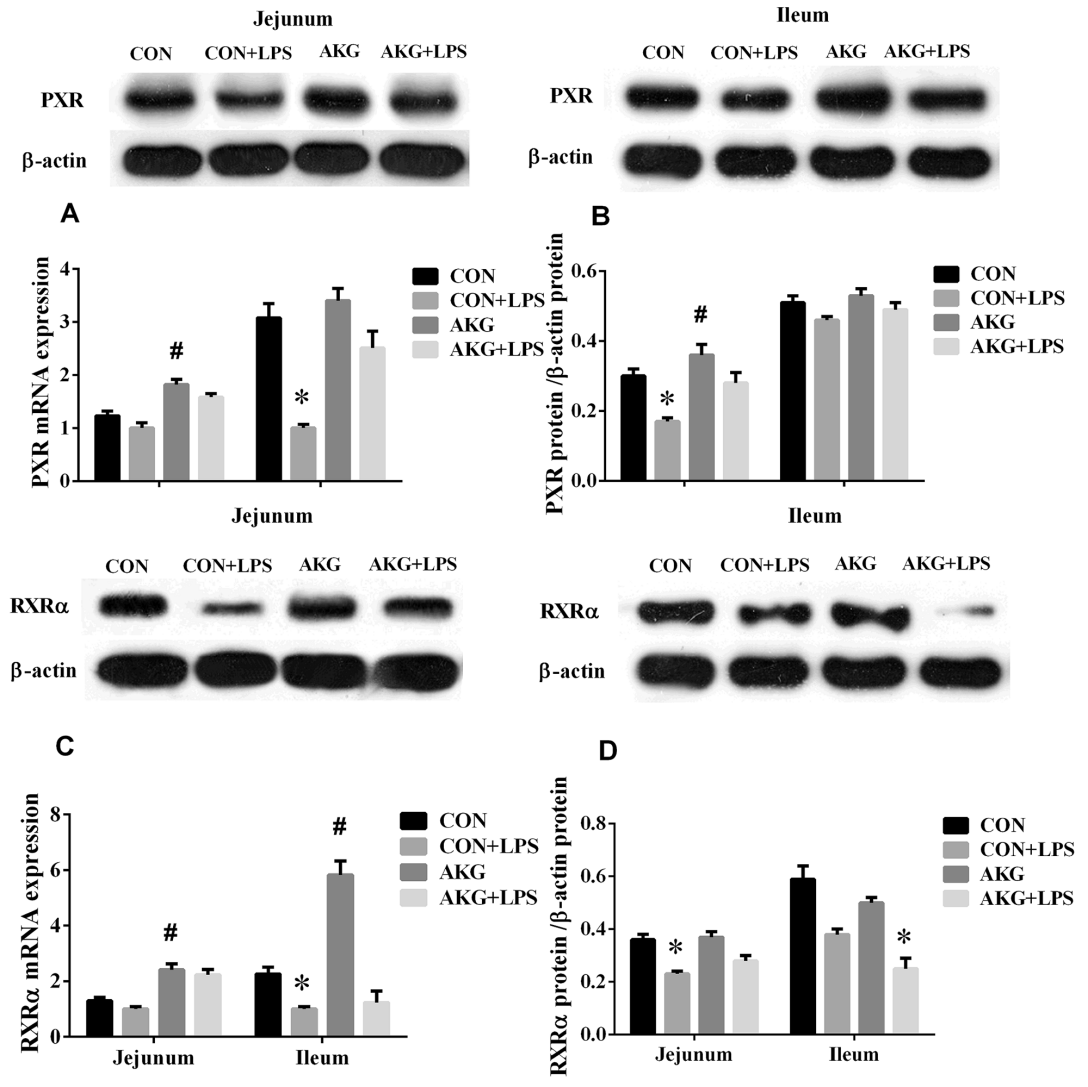
Effects of AKG supplementation on the expression of PXR and RXRα in the jejunum and ileum of weaned piglets

**Figure 6:**
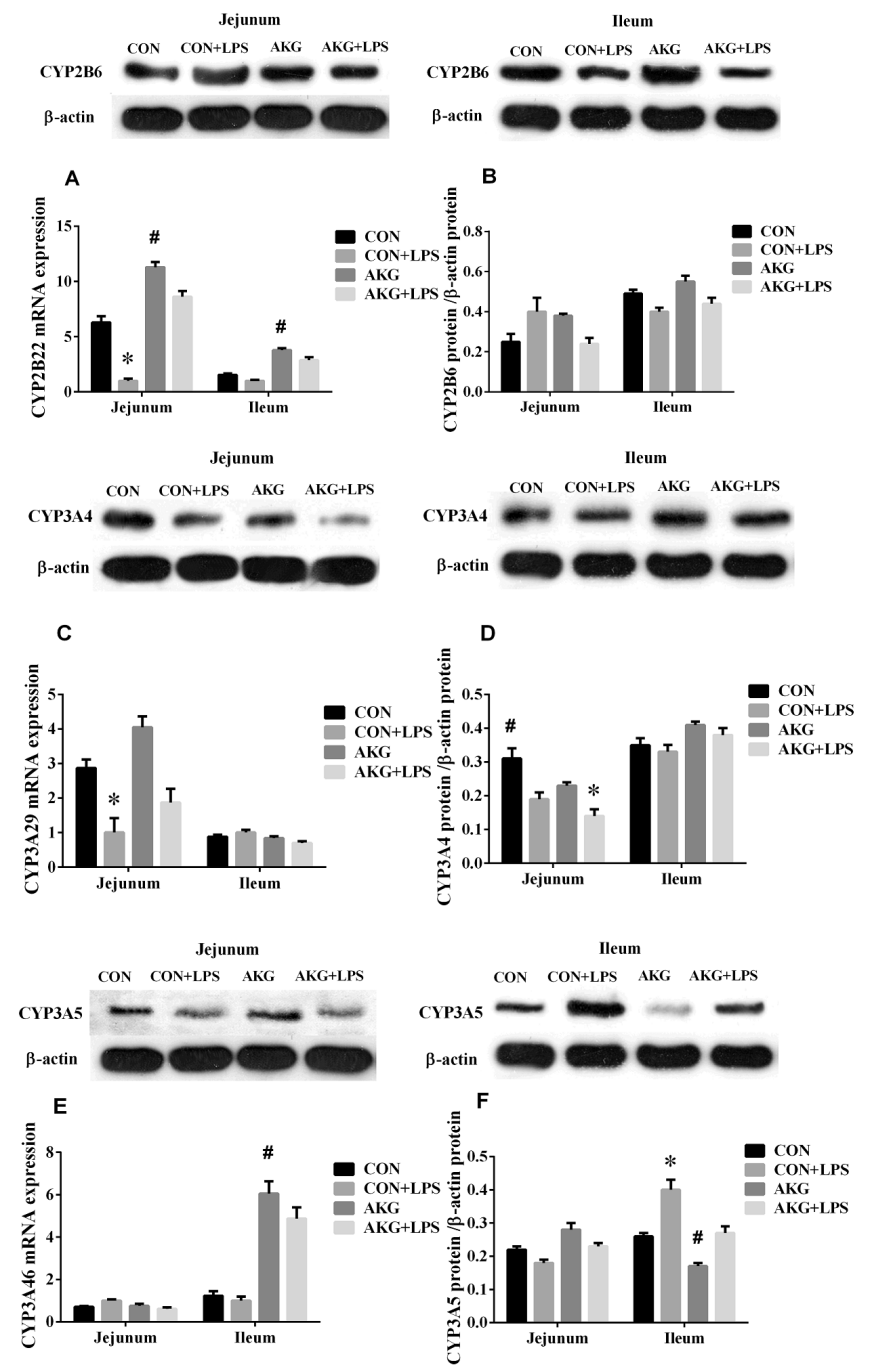
Effects of AKG supplementation on the expression of cytochrome P450 in the jejunum and ileum of weaned piglets

PXR plays an important role in intestinal inflammatory diseases, especially, suppress the activity of NF-κB pathway to alleviate inflammatory response [12]. In our current study, we found that AKG as a nutritional factor, contributed to the activation of PXR signals. Our data showed that, compared with the basal diet, the mRNA expressions of PXR (Figure 5A), RXRα (Figure 5C), and CYP2B22 (Figure 6A) in the jejunum of piglets fed the AKG diet were significantly increased as well as RXRα, CYP2B22, and CYP3A46 (Figure 6E) mRNA in the ileum. However, LPS challenge reduced (P < 0.05) the mRNA abundance of PXR and RXRα, in the ileum of the pigs fed the basal or AKG diet as well as CYP2B22 and CYP3A29 (Figure 6C) in the jejunum. The results indicate that AKG may be enhance the signal of PXR pathway.

**Figure 7:**
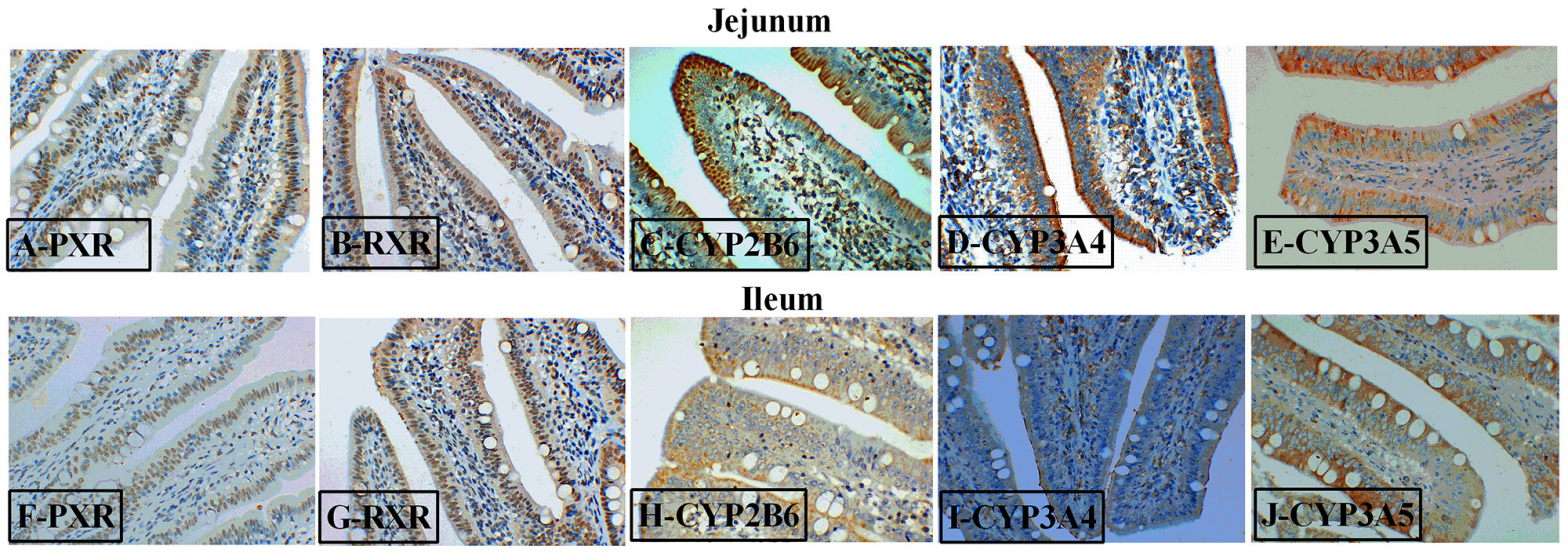
Representative images of immunohistochemical staining (magnification × 400) of the PXR-regulated detoxification pathway in the jejunum and ileum of weaned piglets

To further study the effects of AKG on the PXR pathway, we next examined the key protein expression of PXR-regulated detoxification pathway by Immunohistochemical and Western blot technique in the jejunum and ileum. According to the representative images of immunohistochemical staining (Figure 7), we found thatPXR and RXRa were mostly located in the epithelial cell nucleus of jejunum and ileum, partly in other cell nucleus. However, the CYP450 (CYP2B6, CYP3A4 and CYP3A5) proteins were widely observed in the cytoplasm of epithelial cells in the jejunum and ileum (Figure 7). Interestingly, AKG treatment increased (P < 0.05) the relative expression of PXR protein (Figure 5B) in the jejunum of both saline- and LPS-treated piglets but had no effects (P > 0.05) on those in the ileum. In contrast, LPS challenge decreased the expression of PXR and RXRα (Figure 5D) proteins in the jejunum of piglets fed the basal diet and AKG diet as well as the expression of RXRα protein in the ileum. Furthermore, oral administration with AKG significantly reduced the expression level of CYP3A4 (Figure 6D) protein in the jejunum as well as CYP3A5 (Figure 6F) in the ileum but had no influences on CYP2B6 (Figure 6B) protein level in both the jejunum and ileum of piglets. In addition, LPS challenge decreased the expression level of CYP3A4 protein in the jejunum, while increasing CYP3A5 protein expression in the ileum. This results suggest that AKG has also effect on the downstream expression of PXR-regulated detoxification pathway.

### The activation of PXP pathway by AKG

In the *in vivo* experiment, we found that in the jejunum and ileum, either diets or LPS challenge remarkably influenced the mRNA expression of OGDH by piglets (Figure 3B). AKG supplementation increased the mRNA expression of OGDH by 3.2- and 2.7 –fold in the jejunum and ileum, respectively. To further validate whether AKG supplementation could trigger the PXR-regulated detoxification pathway, we treated intestinal porcine epithelial cells-J2 (IPEC-J2) with rifampicin(RTF) as a comparison in the presence or absence of LPS. Rifampicin could directly activate the activity of PXR and PXR-regulated genes. In IPEC-J2 cells, we found that the addition of AKG could increase the mRNA levels of PXR and its downstream genes such as CYP3As just like as RIF, but no effects was observed for CYP2Bs and RXRα (Figure 3C). Interestingly, the mRNA level of ODGH was also remarkably enhanced by AKG addition.

## DISCUSSION

The nutritional regulation of AKG on improving intestinal immune system has been alluded to briefly in several important studies [9, 13]. In our current study, to screen for this molecular mechanisms that are responsible for the intestinal inflammation in the LPS-challenged piglets, we determined the crosstalk mechanism between the NF-κB-mediated inflammatory pathway and PXR-regulated detoxification pathway mediated by AKG. Firstly, our data showed that AKG supplementation increased or decreased the concentrations of serum biochemical parameters (ALP and IgM), inflammatory cytokines (IL-17, IL-2, and TGF-β), and AKG and its metabolite (*e.g.*, His, Asp, Glu, Gln) in the LPS-challenged piglets, suggesting that AKG is an essential regulator of innate immunity in intestine [9]. The reason might be that LPS as a potential endotoxin, could induce alterations in blood nutrient transport and gastrointestinal metabolism and then result in the decreased intestinal immunity [14, 15], thereby stimulating the rapid synthesis and release of pro-inflammatory cytokine [16]. However, AKG is a key intermediate in amino acid catabolism via phosphate-activated glutaminase, glutamate transaminase and glutamate dehydrogenase [17]. When AKG enters the TCA cycle, it is oxidized by OGDH complex [10, 18–20], leading to producing large amounts of ATP and its metabolites. Thus AKG may bridge cellular energy metabolism and increase amino acid availability to inhibit the secretory of inflammatory cytokines. In addition, these elements involved in AKG and its metabolites can be tied to the enhancement of organic protective metabolism.

It is well-established that toll-like receptors (TLRs) responds to LPS by regulating the expression/activity of NF-κB pathway, whose hyper-activation is mechanistically linked to development of inflammation and immunity disorder in intestinal inflammatory diseases [21]. In keeping with the moderate state of intestinal inflammation, defects observed in LPS-challenged piglets are due to the gross changes in the mRNA expression of TNF-α, TLR4, IL-8 NF-κB, and IκB in the ileum as well as TNF-α, TLR4, IL-10, and IL-17 in the jejunum. Firstly, these differences indicate that the key inflammatory cytokines may persist in a state of elevated stress that may culminate as overt inflammation when exposed to injurious infection [16]. Secondary, the release of several cytokines including TNF-α, IL-10, and IL-17 from activated immune cells occurs a part of the activation of systemic host defense mechanisms [22]. Notably, LPS induced significant activation of the NF-κB-regulated inflammatory pathway with concomitant impairment of intestinal nutrient absorption. Thus, the defense mechanism and the release of inflammatory cytokines mediated by AKG or LPS usually result in the down-regulation of different cytochrome and consuming a large amount of ATP [6]. Zhou et al. reported that TLR2/4 modulated NF-κB-mediated inflammatory pathway in porcine hepatocytes and our results further confirmed it [23]. Our data also supported that AKG supplementation attenuates some inflammatory cytokine generation caused by NF-κB pathway activation in the LPS-challenged piglets.

To further validate our observations regarding on the regulation of AKG on NF-κB-mediated inflammatory pathway in intestinal injury, we evaluated expression levels of NF-κB pathway using the Western blot technique. We found that AKG supplementation decreased the expression of phosphorylated NF-κBp65, IκB, and IKKα proteins in the jejunum and ileum, while increasing the expression of NF-κBp65 and IL-10 proteins, suggesting that AKG administration plays an important role in the suppression of NF-κB pathway. Actually, this regulation is intrinsically associated with intestinal metabolism, specifically metabolism of AKG and its metabolites [9, 24, 25]. Of note, intracellular AKG would likely be almost completely absorbed by intestinal mucosal surfaces thus precluding efficient luminal delivery, then it was utilized in the TCA cycle and generate large amounts of energy in the gut [20].

Recent investigations reported that NF-κB pathway is effectively suppressed by the activation of PXR, which result in the lower expression of pro-inflammatory cytokines (*e.g.* TNF-α, TGF-β, IL-8) [26, 27]. To testify the connection between the NF-κB-mediated inflammatory pathway and PXR-regulated detoxification pathway under AKG treatment in LPS-challenged piglets, we also determined the expression of PXR pathway. Interestingly, we found that that AKG supplementation increased the mRNA expression of PXR and RXRα in the jejunum and ileum of saline- or LPS-treated piglets as well as CYP2B22 and CYP3A46. This suggest that AKG has potent effects in regulating PXR and its downstream targets such as CYP3As and CYP2Bs, although AKG is not a known PXR ligand. To further validate the effect mechanism of AKG on PXR-regulated detoxification pathway, we also treated IPEC-J2 with rifampicin as a comparison, because rifampicin as a PXR ligand could directly activate the PXR activity. The results showed that the addition of AKG also increased the mRNA levels of PXR and its downstream genes such as CYP3As, but no effects was observed for CYP2Bs which is regulated by CAR, and the result is consistent with RIF. Especially, the mRNA level of ODGH was also remarkably enhanced by AKG addition both *in vivo and in vitro*. Collectively, these suggest that once PXR pathway was directly or indirectly mediated by AKG, it will promote the mRNA expression of ODGH in the TCA cycle. Notably, ODGH complex plays a key role in the TCA cycle [28] and is a multienzymatic complex made up of three different types of enzymes, responsible for the conversion of AKG into succinyl CoA and cellular ATP levels [29]. Our previous study demonstrated that AKG might enhance the activity of the AMP-activated protein kinase pathway to defend intestinal inflammation [9] are consistent with our findings and further delineate a possible key mechanism linking PXR to AKG and its metabolites. Moreover, our results also indicate that AKG could improve the activities of key enzymes involved in TCA cycle, then suppressed NF-κB-mediated inflammatory pathway and indirectly enhanced the PXR signals to regulate the activity of CYP450 for achieving self detoxification. In addition, AKG supplementation increased the expression of PXR ligand (RXRα) in the intestine of piglets, which implicated that the activation of PXR may form heterodimers with RXRα under AKG treatment and then regulate the transcription of CYP450 [26].

Since LPS challenge results in increased mucosal permeability and bacterial translocation [16], so it is logical to conclude that the state of activation of intestinal epithelial PXR is paramount in determining the initial events leading to the observed CYP450 expression. The regulatory effect of AKG on the PXR-regulated detoxification pathway in intestinal inflammatory model remain largely unknown. Hence, we attempted to link AKG metabolism to the expression levels and localization of PXR and its downstream targets using the immunohistochemical and Western blot technique. We found thatPXR and RXRa were mostly located in the nucleus of epithelial cell in jejunum and ileum, however, its downstream targets (CYP2B6, CYP3A4 and CYP3A5) were widely observed in the cytoplasm of epithelial cell in the intestine. As noted previously, the NRs often have a similar pattern of tissue distribution [3] and our results further confirmed it.

It has been known that PXR is of critical component of the body’s adaptive defense mechanism against inflammation diseases and verified as an important regulator of CYP450 gene expression [11, 30]. In the current study, the results demonstrate that AKG administration increased the expression of PXR protein in the jejunum and ileum, while decreased the expression of CYP3A4 protein in the jejunum as well as CYP3A5 protein in ileum. These observations have clarified the hypothesis that AKG as a nutritional intervention factor might be similar to those cofactors or ligands in the modulation of PXR-regulated detoxification pathway [11, 31]. Recent studies reported that there was a strong interaction between PXR activation and NF-κB pathway activity [6, 32]. Our data revealed that AKG suppressed the expression of phosphorylated NF-κBp65 and its downstream target (IκB and IKKα) proteins through actions mediated via increasing the PXR-regulated detoxification pathway activity. One potential mechanism for the up-reglulation of the PXR activity is through the down-regulation of NF-κB pathway suppressed by AKG, which in turn de-represses the PXR-regulated gene expression. It is now unclear that whether AKG acts as a regulator to modulate the activity of NF-κB and PXR pathway. However, piglets with LPS infection demonstrate an inverse correlation of the expression of NF-κB and PXR pathway, supporting the view that the anti-inflammatory effect of AKG could improve intestinal health and might provide a potential molecular mechanism via PXR pathway at the interface between detoxification and immune [33]. In addition, one important observation we made is that AKG supplementation increased the expression of IL-10 protein in the jejunum and ileum of both saline- and LPS-treated piglets, indicating that anti-inflammatory cytokine IL-10 was positively regulated by AKG. Pervious studies reported that silent PXR in intestinal epithelial cells decreased the secretory of TGF-β and IL-10, while increasing the expression of TNF-α and IL-8 [34, 35], these observations are consistent with our results.

Interestingly, PXR-regulated detoxification pathway modulated by AKG may be intrinsically linked to intracellular AKG metabolism as well as to the inhibition of NF-κB, however, further investigation are warranted to obtain more evidence to examine these possible mechanisms. Many studies reported that the expression variation of CYP450 could in turn cause changes in detoxification and metabolic bioactivation in inflammatory tissues [36, 37]. Moreover, the ability of inflammatory mediators (*e.g.,* IL-6, TNF-α) to suppress the expression of CYP450 (*e.g.*, CYP3As, CYP2Bs) could affect the homeostasis of intestinal health [23]. Some mechanisms widely proposed that the crosstalk between NF-κB and PXR pathway could regulate the expression of CYP450 [23, 38, 39]. The increased activity of PXR-regulated detoxification pathway and the reduced expression of NF-κB-mediated inflammatory pathway according to our results are consistent with these findings. Furthermore, we also found that phosphorylation of NF-κBp65 (Ser536) were remarkably decreased by AKG treatment, and the addition of AKG had different effects on the expression of different CYP450 (the most important family of drug metabolizing enzymes). These results suggest that exogenous and endogenous AKG combined with its metabolites (i.e., Asp, Glu, Gln) and ODGH complex contribute to initiation of cell signaling in the PXR and NF-κB pathway, which mediates the transcription of CYP450 and the secretory of inflammatory cytokines to enhance intestinal immunity.

**Figure 8:**
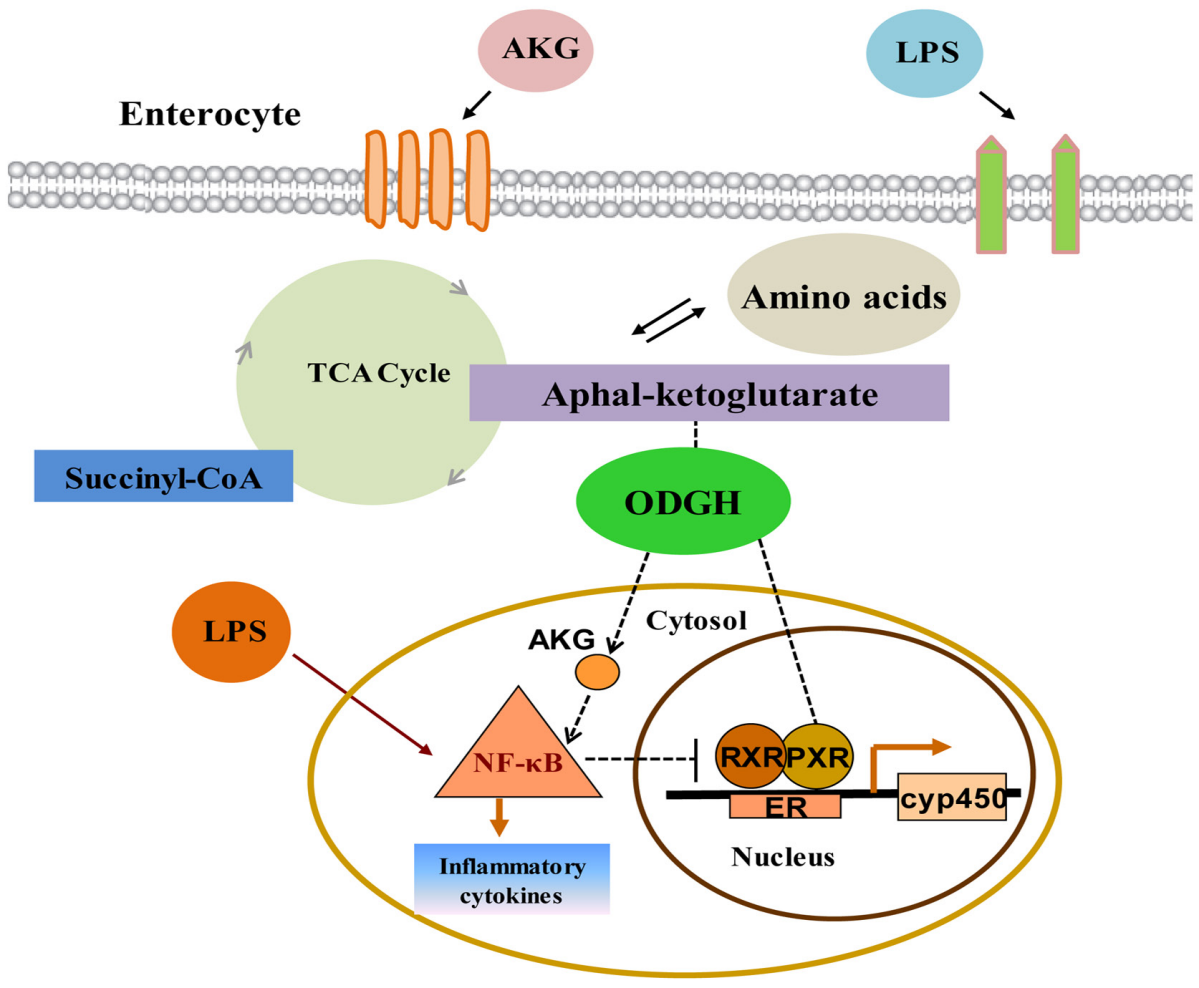
Interactions between PXR and NF-κB pathway modulated by AKG in intestine LPS triggers the activation of NF-κB-mediated inflammatory pathway and promotes the secretion of inflammatory cytokines. AKG supplementation facilitates intracellular AKG metabolism with concomitant absorption and transport of intestinal nutrient, which co-administrate to enhance the expression of ODGH in the TCA cycle and generate large amounts of ATP for maintaining gut homeostasis. Thus AKG could reverse the adverse effects induced by LPS and inhibit the NF-κB-mediated inflammatory pathway activity, which then would directly or indirectly enhance PXR signal, although AKG is not a known PXR ligand. One potential mechanism for the up-regulation of the PXR-regulated detoxification pathway is through the down-regulation of NF-κB pathway by AKG interaction which in turn de-represses the PXR-associated gene and protein expression, thereby improving intestinal immunity. ER: endoplasmic reticulum.

In conclusion, these observations provide important molecular biology steps toward a more comprehensive understanding of interactions between PXR and NF-κB pathway by AKG modulation (Figure 8). More importantly, this is a new discovery to systemically investigate the roles of AKG on the interaction between PXR-regulated detoxification pathway and NF-κB-mediated inflammatory pathway, and it will provide a new practical solution to solve a range of intestinal inflammatory diseases. These novel findings have important implications for the development of nutritional interventions to ameliorate livestock production and human health.

## MATERIALS AND METHODS

### Animals and experimental design

This study was approved by the animal welfare committee of the Institute of Subtropical Agriculture, Chinese Academy of Sciences (2013020; Changsha, China). Thirty-two cross-bred (Duroc ×Landrace ×Yorkshire; average body weight (BW) = 6.24 ± 0.11 kg) piglets were weaned at 28 days, and were randomly assigned to either a basal (CON) or basal+1% alpha-ketoglutarate (AKG) diet (n=16/diet, 8 male and 8 female). Then each group of weaned pigs was divided randomly into two sub-groups (n=8/treatment group, CON+LPS and AKG+LPS), which were fed their respective diets and ad libitum. At 10:00 am on days 22, 25, 28 and 30, piglets in the CON+LPS and AKG+LPS groups were intraperitoneally injected with *E.coli* LPS at 100μg/kg BW), respectively, whereas pigs in the CON and AKG groups were injected intraperitoneally with the same volume of sterile saline. The composition and nutrient levels of the diets met the nutrient specifications for 5 to 10 kg BW pig were described by our previous study [10]. The dosage of alpha-ketogrurate (Wuhan Yuancheng Gongchuang Technology co., LTD, Wuhan, China; purity ≥ 99.2%) as well as LPS( *Escherichia coli* serotype 055:B5; Sigma Chemical, Inc., St Louis, MO, USA), were adopted according to the previous studies [10, 40].

### Sample collection

On day 30, all pigs were anaesthetized with an intravenous injection of sodium pentobarbital (50 mg/kg BW), then 10 mL of blood was collected aseptically in tubes from a jugular vein after LPS challenge. Serum samples were obtained by centrifugation at 3000 × g for 10 min at 4 °C, and then immediately stored at -80 °C for subsequent determination of serum biochemical parameters and free amino acids. The small intestine was dissected free of the mesentery and sampled on a chilled stainless-steel tray. Segments (10 cm) were collected from the middle jejunum and distal ileum, respectively, and thoroughly flushed with ice-cold phosphate-buffered saline solution, then immediately frozen in liquid nitrogen and then stored at -80 °C for the analysis of RNA extraction and western blot analysis. And one segment was fixed in 10 % neutral buffered formalin for immunohistochemical analysis.

### Cell culture

Intestinal porcine epithelial cells-J2 (IPEC-J2) isolated from neonatal piglet mid-jejunum, were used to investigate the effects of AKG on the expression of PXR-regulated detoxification pathway according to previous report [41]. IPEC-J2 cells were grown in serial passage in uncoated plastic culture flasks in DMEM-H containing 10% FBS, 5 mM l-glutamine, 100 U/ml penicillin, and 100 μg/ml streptomycin. At confluence, cells were trypsinized and seeded in 96, 24, or 6 well cell culture plates with approximately 30-40% cells per well and maintained at 37°C in a 5% CO_2_ incubator. After an overnight incubation, cells were incubated with AKG-free medium containing 0, 2 mM AKG or 0, 10μM rifampin (RIF) for 48 h and then cells were challenged with 0 or 20 ng/ml LPS. At the end of a 24h culture period, the medium was collected and cells were rapidly washed three times with 2 ml ice-cold PBS and were collected for further research.

### Determination of blood biochemical parameters and amino acid profile

Serum biochemical parameters, including total protein (TP), total albumin (ALB), alkaline phosphatase (ALP), and immunoglobulin M(IgM), were measured using spectrophotometric kits (Nanjing Jiangcheng Biotechnology Institute, Nanjing, Jiangsu, China) in accordance with the manufacturer’s instructions. Serum amino acids(Arg, His, Glu, Gln, Pro, AKG) were determined by an automatic amino acid analyzer (L-8900; Hitachi Global Inc., Hitachi, Japan) as described previously [42].

### Analysis of serum inflammatory cytokines

Serum physiological concentrations of IL-2, IL-8, IL-17, and TGF-β were determined using ELISA test kits (Wuhan Huamei Biotech co., LTD, Wuhan, Hubei, China) in accordance with the manufacturer’s instructions.

### Quantification of mRNA and cDNA synthesis by real-time PCR analysis

The primers were designed with the use of Primer 5.0 according to the gene sequence of pigs to produce amplification products (Table 1). The mRNA expression of gene in the jejunum and ileum was analyzed by Real-time PCR as described previously [43]. Relative gene expression was expressed as a ratio of the target gene to the control gene using the formula 2^-(ΔΔCt)^, where ΔΔCt = (Ct_Target_ – Ct_β-actin/GAPDH_)_treatment_ – (Ct_Target_ – Ct_β-actin/GAPDH_)_control_.

**Table 1:** Primers used for quantitative reverse transcription-PCR

Accession no.	Gene	Primers	Product length(bp)
XM_003124280.3	β-actin	F:CTGCGGCATCCACGAAACT	147
		R:AGGGCCGTGATCTCCTTCTG	
NM_001206359.1	GAPDH	F: AAGGAGTAAGAGCCCCTGGA	140
		R: TCTGGGATGGAAACTGGAA	
NM_001038005.1	PXR	F: ATTGATTTGCGTGGATGCTGAACTG	191
		R:TGTAGTCCCAGTATTCCAGCCTCG	
XM_001927453.2	RXRα	F:CAAGTGCCTGGAACACCTCT	240
		R:ATGGAAGGTAACAGGGTGGC	
		R: AGAGGACCTGCTGCTTGTTC	
NM_214413.1	CYP2B22	F:GGGAACGTTGGAAGACCCTT	228
		R:CGGGATCTCTGTAGGCGAAG	
NM_214423.1	CYP3A29	F:CCTGAAATTAACCACGCAAGGGCT	140
		R:TCTGGGATGCAGCTTTCTTGACCA	
NM_001134824.1	CYP3A46	F:GCTGCATCCCAGAGTACCAG	199
		R:AGAAGCTGAGTCTGCATGTCTG	
XM_003134891.5	OGDH	F:CGACCAGAACGTGGACAAGA	189
		R:GCCGTGTTGTGAAAGTCACC	
NM_001048232.1	NF-κB	F:AGCCATTGACGTGATCCAGG	248
		R:CGAAATCGTGGGGCACTTTG	
XM_001924394.4	IκB	F:CACCCGAGTTAGAAGGGCTC	155
		R:GGTATCTGCTGAGGTGTGCTG	
		R:TCAGCGAAGGTGTCATTATTGC	
NM_001113039.2	TLR4	F:GCCATCGCTGCTAACATCATC	108
		R:CTCATACTCAAAGATACACCATCGG	
NM_214022.1	TNF-α	F:CCACGTTGTAGCCAATGTCA	395
		R:CAGCAAAGTCCAGATAGTCG	
NM_213867.1	IL-8	F:AGAACTGAGAAGCAACAACAACAG	131
		R:CACAGGAATGAGGCATAGATGTAG	
NM_214041.1	IL-10	F:ATGGGCGACTTGTTGCTGAC	217
		R:CACAGGGCAGAAATTGATGACA	
NM_001005729.1	IL-17	F:CTCTCGTGAAGGCGGGAATC	137
		R:GTAATCTGAGGGCCGTCTGG	

### Immunohistochemical analysis

The protein expressions of nuclear receptor (PXR, RXRα) and cytochrome P450 (CYP2B6, CYP3A4, and CYP3A5) in jejunum and ileum of piglets were determined using immunohistochemical analysis as described by Wang et al. [44]. Sections were incubated with the anti PXR (1:50; Proteintech Group, Inc., Los Angeles, CA, United States), RXRα (1:150; Proteintech Group, Inc.), CYP2B6 (1:100; Proteintech Group, Inc.), CYP3A4 (1:50; Proteintech Group, Inc.), CYP3A5 (1:100; Proteintech Group, Inc.). The protocol was described by Wang et al. [44] and the stained sections were scored independently by 2 investigators using a microscope at 400-fold magnification (Olympus, Tokyo, Japan).

### Western blot analysis

Jejunum and ileum were extracted with total protein extraction reagents (Thermo Fisher Scientific Inc., New York, NK, USA) in accordance with the manufacturer’s instructions. The relative expression of protein was determined by Western blot technique as described previously [22]. The following antibodies were used for protein quantification: PXR (1:500; Proteintech Group, Inc.), RXRα (1:500; Proteintech Group, Inc.), CYP2B6 (1:200; Proteintech Group, Inc.), CYP3A4 (1:500; Proteintech Group, Inc.), CYP3A5 (1:2000; Proteintech Group, Inc.), NF-κBp65 (1:1000; Cell Signaling Technology, Danvers, MA, USA), phosphorylated NF-κBp65 (Ser536) (1:1000; Cell Signaling Technology, Danvers, MA, USA), IKKα(1:1000; Santa Cruz Biotechnology, Dallas, TX, USA), IκB (1:1500; Proteintech Group, Inc.), IL-10(1:1000; Abcam, Cambridge, LON, UK) and β-actin (1:4000;Proteintech Group, Inc.)and secondary antibody horseradish peroxidase-conjugated goat anti-rabbit IgG (1:6000; Proteintech Group, Inc.) or anti-mouse IgG (1:4000; Proteintech Group, Inc.). All protein measurements were normalized to β-actin (1:4000; Proteintech Group, Inc.) and data are expressed relative to the values in control piglets. In addition, porcine protein CYP2B22, CYP3A29, CYP3A46 have a high homology similarity (over 90 percent) with human protein CYP2B6, CYP3A4, CYP3A5, respectively.

### Statistical analysis

All statistical analyses were performed by one-way ANOVA or factorial ANOVA using a mixed procedure (PROC MIXED) of SAS software version 9.2 (SAS Institute Inc., Cary, NC, USA). The statistical model included the effects of challenge (saline or LPS), diet (basal or AKG), and their interactions. All data were presented as Least Squares means plus pooled SEM. The Tukey multiple comparison test was used to evaluate the differences among the treatments. Probability values ≤ 0.05 were taken to indicate statistical significance.
